# In Vitro Biofilm Formation on Zirconia Implant Surfaces Treated with Femtosecond and Nanosecond Lasers

**DOI:** 10.3390/jfb14100486

**Published:** 2023-09-22

**Authors:** Soo Kyum Bihn, Keunbada Son, Young-Tak Son, Ram Hari Dahal, Shukho Kim, Jungmin Kim, Jun Ho Hwang, Sung-Min Kwon, Jong Hoon Lee, Hyun Deok Kim, Jae-Mok Lee, Myoung-Uk Jin, Kyu-Bok Lee

**Affiliations:** 1 Department of Prosthodontics, School of Dentistry, Kyungpook National University, Daegu 41940, Republic of Korea; nicejiya527@naver.com; 2 Advanced Dental Device Development Institute (A3DI), Kyungpook National University, Daegu 41940, Republic of Korea; oceanson@knu.ac.kr (K.S.); dudxkr741@naver.com (Y.-T.S.); 3 Department of Dental Science, Graduate School, Kyungpook National University, Daegu 41940, Republic of Korea; 4 Department of Microbiology, School of Medicine, Kyungpook National University, Daegu 41944, Republic of Korea; ramhari.dahal@knu.ac.kr (R.H.D.); shukhokim@knu.ac.kr (S.K.); minkim@knu.ac.kr (J.K.); 5 Institute of Advanced Convergence Technology, Kyungpook National University, Daegu 41061, Republic of Korea; hjh@iact.or.kr (J.H.H.); sungmin@iact.or.kr (S.-M.K.); laser@knu.ac.kr (J.H.L.); 6 School of Electronics Engineering, Kyungpook National University, Daegu 41566, Republic of Korea; hdkim@knu.ac.kr; 7 Department of Periodontology, School of Dentistry, Kyungpook National University, Daegu 41940, Republic of Korea; leejm@knu.ac.kr; 8 Department of Conservative Dentistry, School of Dentistry, Kyungpook National University, Daegu 41940, Republic of Korea

**Keywords:** zirconia dental implant, femtosecond laser, nanosecond laser, biofilm formation

## Abstract

(1) Background: The purpose of this study was to evaluate how a zirconia implant surface treated with laser technology affects the degree of biofilm formation. (2) Methods: Experimental titanium (Ti) disks were produced that were sandblasted with large grit and acid-etched (T), and they were compared with zirconia (ZrO_2_) discs with a machined (M) surface topography; a hydrophilic surface topography with a femtosecond laser (HF); and a hydrophobic surface topography with a nanosecond laser (HN) (N = 12 per surface group). An in vitro three-species biofilm sample (Aggregatibacter actinomycetemcomitans (Aa), Porphyromonas gingivalis (Pg), Prevotella intermedia (Pi)) was applied to each disc type, and bacterial adhesion was assessed after 48 and 72 h of incubation using an anaerobic flow chamber model. Statistical significance was determined using the Kruskal–Wallis H test, with Bonferroni correction used for the post-hoc test (α = 0.05). (3) Results: Compared to the T group, the M group exhibited more than twice as many viable bacterial counts in the three-species biofilm samples (*p* < 0.05). In comparison to the T group, the HF group had significantly higher viable bacterial counts in certain biofilm samples at 48 h (Aa and Pi) and 72 h (Pi) (*p* < 0.05). The HN group had higher viable bacterial counts in Pi at 48 h (5400 CFU/mL, *p* < 0.05) than the T group (4500 CFU/mL), while showing significantly lower viable bacterial counts in Pg at both 48 (3010 CFU/mL) and 72 h (3190 CFU/mL) (*p* < 0.05). (4) Conclusions: The surface treatment method for zirconia discs greatly influences biofilm formation. Notably, hydrophobic surface treatment using a nanosecond laser was particularly effective at inhibiting Pg growth.

## 1. Introduction

Titanium (Ti), recognized for its excellent biostability, biocompatibility, mechanical performance, and long-term durability, is extensively utilized in dentistry [1,2,3,4,5]. However, zirconia (ZrO_2_) has emerged as a popular alternative to titanium in dental implantology [6]. Zirconia has a good bone reaction, can minimize inflammatory reactions to soft tissues adjacent to the surface, is biocompatible and aesthetic, has low bacterial attachment, and has high fracture strength and axial strength [7]. The type of zirconia currently used in dentistry includes yttrium-stabilized tetragonal zirconia (Y-TZP), containing more than 90% zirconium dioxide and glass-injected ceramics and more than 35% partially stabilized zirconia. Due to the excellent mechanical properties of Y-TZP ceramics, they are currently widely used in the dental field. They are subjected to surface sandblasting. [8]. In the past, various laser treatment methods have been used for zirconia surface treatment, but these methods have caused unexpected reactions, such as forming debris on the surface and changing physical properties [9]. Despite these advancements, a loss rate of 7% has been reported in the 10-year survival rate of implants [10]. Such losses predominantly occur due to procedural errors, infections, or premature loading, with the majority of implant losses largely being due to the loss of bone support [10].

The formation of biofilms around the implant can significantly contribute to peri-implantitis [11]. Certain bacterial strains play significant roles in this respect. Clinical studies of microorganisms around implants with peri-implantitis report that Gram-negative anaerobic bacteria mainly exist at high rates and in high numbers in the mucosal junction of implants. Specifically, *Aggregatibacter actinomycetemcomitans* (Aa) is a Gram-negative bacterium that predominantly contributes to aggressive periodontitis, whereas *Porphyromonas gingivalis* (Pg) and *Prevotella intermedia* (Pi), both Gram-negative anaerobes, are implicated at the beginning of chronic periodontitis and peri-implantitis [11,12,13].

The success of implants heavily relies on effective tissue adhesion, which in turn is influenced by the surface topography and wettability of the implant [13,14]. Various methods have been suggested to modify the surface structure of implants. However, many of these have resulted in surface defects that negatively impact long-term prognosis. A recent study suggests that laser treatment on implant surfaces offers a more optimal surface structure and superior control over contamination compared to other methods [15]. Surface treatment using a laser is a technique capable of forming a uniform structure on a surface. Previous studies have shown that by forming micro-structured roughness through lasers, hMSCs can enhance osteoblast differentiation [16,17] and in vivo bone adhesion [18,19]. We observed surface roughness to evaluate the surface formed by a laser. There are various methods for measuring surface roughness, but among them, Ra (arithmetical mean height) and Rz (maximum height of profile) values are obtained, and Ra is the most commonly used method for measuring surface roughness.

In particular, femtosecond and nanosecond lasers have emerged as significant tools in surface treatment, providing high accuracy, minimizing heat generation, and leaving minimal debris on the implant surface [15,20]. There have been many efforts to improve bone production performance, among which femtosecond laser technology appears to be a promising surface treatment technique that controls the adhesion, diffusion, and movement of hMSCs. Many studies have reported surface treatment using long-pulse lasers such as nanosecond Nd:YAG lasers [21], copper vapor lasers [22], nanosecond excimer lasers [23], picosecond ND:YAG lasers [24], and sub-picosecond excimer lasers [25]. While in vitro studies have demonstrated the influence of surface properties such as roughness, surface free energy, wettability, and sterilization on biofilm formation and bacterial distribution [26,27], there remains a scarcity of research examining the impact of different laser systems on biofilm formation on implant surfaces. Various studies have demonstrated that the use of femtosecond laser technology can form nano-textured grooves [16] and grids [17]. A previous study evaluated the formation of biofilms by treating the surface of titanium implants using femtosecond and nanosecond lasers and changing the surface modification to hydrophilic and hydrophobic properties [28]. However, there is still a lack of research using femtosecond and nanosecond lasers to modify the surface of ceramic implants and evaluate dental biofilm growth.

Previous in vitro studies of implant surfaces studied the formation of biofilm using samples such as disks or slabs containing the surface to be studied [29]. In this study, a disk with a diameter of 10 mm and a thickness of 5 mm was produced to observe the formation of biological membranes for 48 h and 72 h.

Therefore, the purpose of this study was to examine the impact of various surface treatments (machined, hydrophilic, and hydrophobic) on zirconia disc surfaces on the formation of three-species biofilms after 48 and 72 h, compared to sandblasted large-grit and acid-etched (SLA) titanium surfaces.

The null hypothesis of this study was that the method of zirconia implant surface treatment would not lead to any significant difference in viable bacterial counts following biofilm formation, nor would it show any significant differences when compared to SLA-treated titanium surfaces.

## 2. Materials and Methods

To determine the number of samples per group, 5 pilot experiments were performed prior to the present study, and 12 per group were determined based on the following results using a power analysis software (G*Power v3.1.9.2; Heinrich Heine University, Dusseldorf, Germany): effect size (f) = 0.52; power = 99%; actual power = 99.20%.

To prevent deviations according to the investigator, experienced researchers conducted repeated experiments under the same conditions, and all devices used were calibrated according to the manufacturer’s instructions before use. In addition, all experiments were conducted under the same conditions.

### 2.1. Specimen Fabrication

Disks with a diameter of 10 mm and thickness of 5 mm were produced and underwent SLA surface treatment, as performed by a dental implant manufacturer (DENTIS; Daegu, Republic of Korea) (N = 12 per surface treatment group). The composition of the Ti disks (grade 4) included 0.08% carbon (C), 0.5% iron (Fe), 0.015% hydrogen (H), 0.05% nitrogen (N), 0.40% oxygen (O), and 98.9% Ti. The zirconia disks were solely composed of ZrO_2_ (Y-TZP) without any other additives. The zirconia consisted of 93.0 wt% ZrO_2_, 5.0 wt% Y_2_O_3_, 0.1 wt% Al_2_O_3_, and 1.9 wt% HfO_2_, with a grain size of 0.3 µm. Zirconia disks without any surface treatment were classified as the machined group (M group).

Titanium disks underwent sandblasting via a sandblasting with large-grit alumina particles for 20 s under 4 bar pressure. Subsequently, they were acid-etched using a mixture of HCL and H_2_SO_4_, diluted with distilled water, and air-dried at room temperature. The resulting specimens were categorized as the titanium SLA surface group (T group).

According to the previous study, we determined the desired form and the parameters [17]. In this study, a custom femtosecond laser system with very short pulses was used for surface treatment of the zirconia disks. Using this system, a linear pattern with 50 µm grooves was formed on the zirconia disk surface at a speed of 10 mm per second using a laser with a wavelength of 343 nm. These zirconia disks were categorized as the hydrophilic with a femtosecond laser group (HF group).

Using a custom nanosecond laser system, a grid pattern with 200 µm grooves was formed on the zirconia disk surface at a speed of 100 mm per second using a laser with a wavelength of 355 nm. These zirconia disks were categorized as the hydrophobic with a nanosecond laser group (HN group).

In the present study, specimens with all surface treatments completed were cleaned and sterilized by the implant manufacturer (DENTIS; Daegu, Republic of Korea) and were packaged to prevent contamination.

### 2.2. Surface Wettability

The wetting properties of the treated samples were assessed using a contact angle goniometer (Phoenix-MT; SEO, Suwon, Republic of Korea). To carry this out, a droplet of water measuring 2 μm was deposited onto each test surface with a micro-syringe. The angle of contact between the droplet and the surface was recorded within a span of 10 s. An average contact angle was obtained by analyzing five readings for each individual sample.

### 2.3. Scanning Electron Microscopy and Confocal Scanning Microscopy

Firstly, each disk was purified through an ultrasonic cleaning process that included a balanced 1:1:1 mixture of acetone, ethanol, and distilled water; this lasted for 15 min. Following this, the disks were left to dry at 60 °C for two hours. After drying, the titanium disks were coated thinly with gold-palladium (HPC-1SW; Vacuum Device Inc., Mito, Japan) for observation purposes. Scanning electron microscopy (SEM; Hitachi SU8230; Hitachi, Tokyo, Japan) was then employed at an acceleration voltage of 5 kV to capture images at both 100× and 500× magnifications.

To obtain confocal images, we used a laser scanning confocal microscope (LEXT OLS4100; Olympus, Tokyo, Japan) at a magnification setting of 10×. From these captured images, the roughness indicators—specifically the arithmetical mean height (Ra) and the Rz maximum height of profile (Rz)—were ascertained for each material type. These calculated values were then employed for conducting a comparative assessment between the different surface treatment methods applied.

### 2.4. Bacterial Strains and Culture Conditions

In a previous study evaluating biofilm formation on implant surfaces, experiments were conducted using modified BHI when culturing biofilm [30]. Experiments were conducted using three bacterial strains: Aa, Pg, and Pi. All strains were cultured under anaerobic conditions comprising 5% CO_2_, 5% H_2_, and 90% N_2_ in a modified brain–heart infusion enriched with yeast extract, L-cysteine HCL, hemin solution, and vitamin K3, and incubated at 37 °C for 24–48 h. For agar plates, 1.5% agar and 5% defibrinated sheep blood were added.

### 2.5. Biofilm Development and Bacterial Viability Assay

For biofilm formation, both Ti and zirconia disks were cultured in 2 mL of media containing Aa, Pg, or Pi bacteria in a 24-well culture plate. The media were incubated in an anaerobic environment with 10% H_2_, 10% CO_2_, and balance N_2_ at 37 °C for both 48 and 72 h.

After 48 and 72 h of incubation, the disks were rinsed three times with phosphate-buffered saline. To kill the bacteria on the bottom side of each disk, one-third of the surface was rinsed with 70% alcohol. These alcohol-rinsed sections were further rinsed three times with phosphate-buffered saline. Each disk was then placed in a sealed sterilization pouch and subjected to ultrasonic treatment for 5 min to detach the bacteria, after which they were transferred to a 12-well culture plate for biofilm evaluation. The samples were then diluted in 15 mL of PBS solution, placed on agar plates, and cultured anaerobically at 37 °C for 72 h. The CFU is an indicator used to evaluate the growth of bacteria or microorganisms and is a method of measuring the number of visible bacteria or fungi. In this study, we used the CFU to compare the number of bacteria grown on the disk surface. The number of colony-forming units (CFUs) was calculated, and viable bacteria were identified.

### 2.6. Statistical Analysis

The sample size for this study was determined through a power analysis (G*Power version 3.1.9.2; Heinrich Heine University, Dusseldorf, Germany) to ensure adequate power to detect statistically significant differences between the treatment groups. Statistical analyses were performed using statistical software (SPSS version 26; IBM Corp., Armonk, NY, USA). Due to the results of normality tests, non-parametric statistical methods were adopted. Statistical significance among the four surface treatments was determined using the Kruskal–Wallis H test (α = 0.05). In terms of post-hoc tests, we employed the Bonferroni correction for validation (α = 0.05).

## 3. Results

Variations in surface contact angles were observed based on different implant surface treatment methods (Table 1) (*p* < 0.001). The T and HF groups showed similar contact angles (*p* > 0.05), while the HN group displayed the highest contact angle (Table 1) (*p* < 0.05).

**Figure 1:**
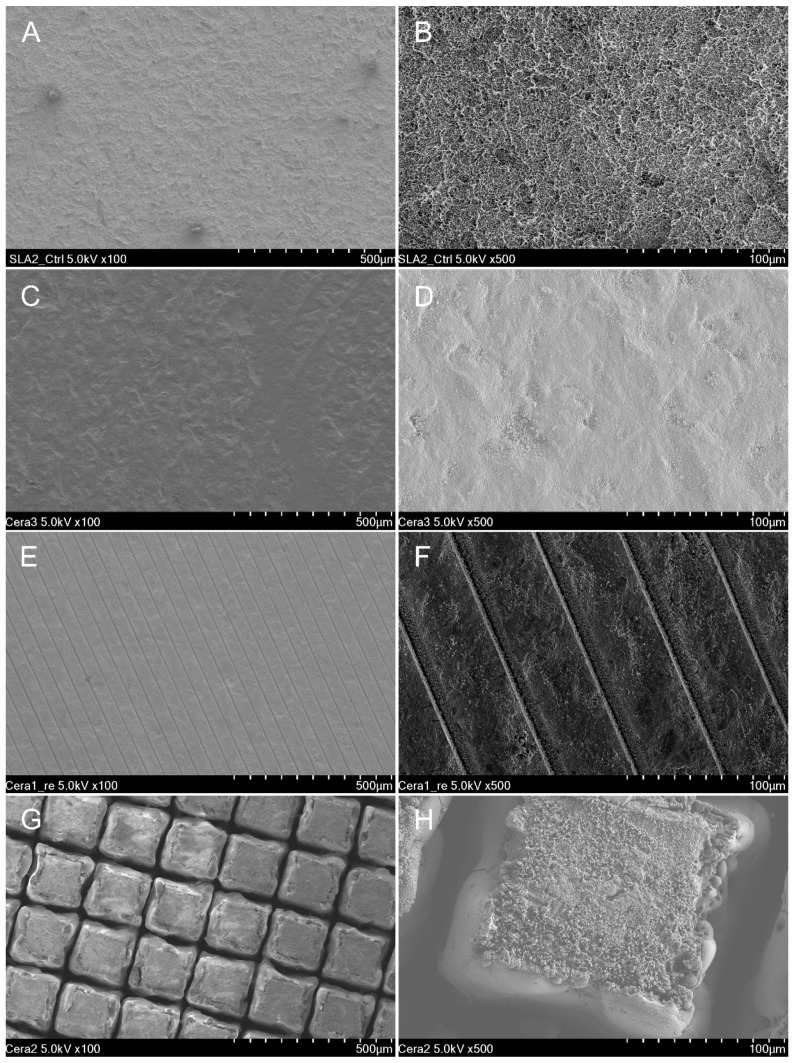
Representative surface morphology image using SEM at 100× and 500× magnification. (**A**,**B**): T group. (**C**,**D**): M group. (**E**,**F**): HF group. (**G**,**H**): HN group. (**A**): Reprinted with permission from Ref. [28].

The sample surface morphologies in SEM images were examined post-disc surface treatment for each group (Figure 1). The T group demonstrated micro-pits formed on the mechanically processed titanium disc, achieved by sandblasting and acid etching (Figure 1A,B). The M group exhibited a smooth zirconia disc surface with visible machining marks (Figure 1C,D). The HF group showed consistent and repeated parallel lines formed on the zirconia disc through the use of femtosecond lasers to enhance hydrophilicity (Figure 1E,F). In contrast, the HN group revealed a repetitive lattice pattern on the zirconia disc, created using nanosecond lasers to augment hydrophobicity (Figure 1G,H).

**Figure 2:**
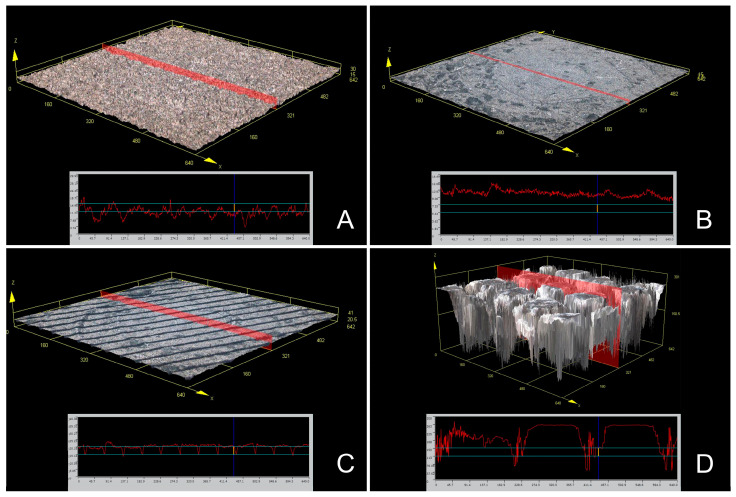
Representative surface morphology image using confocal laser scanning microscopy. (**A**): T group. (**B**): M group. (**C**): HF group. (**D**): HN group.

Surface roughness and sample surface morphology were observed in each group using confocal scanning microscopy (Figure 2). The observed sample surface morphologies were consistent with the SEM results (Figure 2). Significant variations in disc surface roughness (Ra, Rz) were noticed based on the surface treatment group (Table 2) (*p* < 0.001). The HN group displayed a notably higher surface roughness (Ra, Rz) than all other groups (Table 2) (*p* < 0.05). Both the T and HF groups exhibited similar surface roughness levels (Ra, Rz) (Table 2) (*p* > 0.05).

Regarding the number of surviving bacteria (CFU/mL) in the biofilms formed over 48 h, significant differences were observed according to the disc surface treatment group (Table 3) (*p* < 0.001). Biofilms of Aa, Pg, and Pi all showed variations based on the surface-treated group (Table 3) (*p* < 0.001). In Aa, the T and HF groups showed the fewest bacteria, while the M group had the most (Table 3) (*p* < 0.05). For Pg, the HN group had the fewest bacteria, and the M group displayed the highest number (Table 3) (*p* < 0.05). In Pi, the T group presented the fewest bacteria, with the M group showing the most (Table 3) (*p* < 0.05).

Significant differences in the number of surviving bacteria (CFU/mL) in biofilms formed over 72 h were observed depending on the disc surface treatment group (Table 4) (*p* < 0.001). Biofilms of Aa, Pg, and Pi all exhibited differences based on the disc surface-treatment group (Table 4) (*p* < 0.001). In Aa and Pi, the T and HF groups displayed the fewest bacteria, while the M group showed the most (Table 4) (*p* < 0.05). In Pg, the HN group revealed the fewest bacteria, with the M group exhibiting the highest bacteria count (Table 4) (*p* < 0.05).

When comparing the CFU counts between 48 and 72 h, most disc surface-treatment groups displayed a significant increase in bacterial numbers at 72 h (Table 4) (*p* < 0.05). However, in Pg for the T group (*p* = 0.092) and in Pi for the HN group (*p* = 0.164), no significant bacterial increase was observed at 72 h (Table 4) (*p* > 0.05).

**Figure 3:**
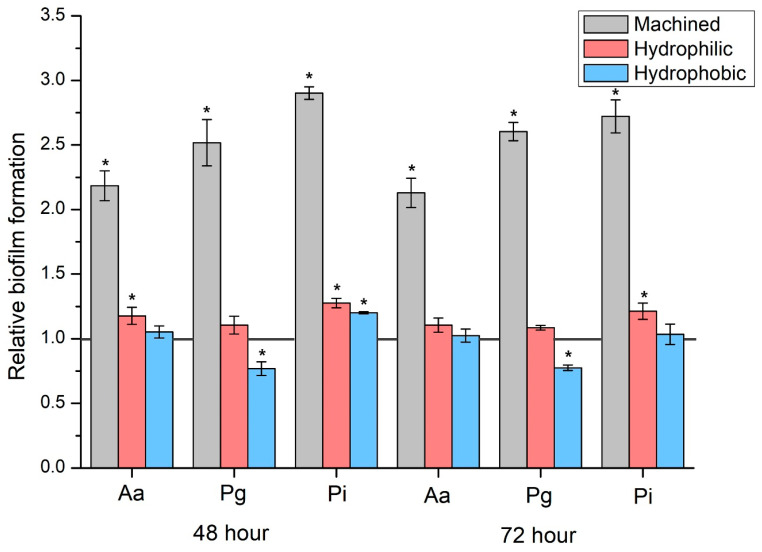
Comparison of relative biofilm formation ratios in relation to the T group. * Significant difference when compared with the T group determined using the Mann-Whitney U test, *p* < 0.05.

When comparing the zirconia disc groups against the T group as a reference, the M group displayed over a two-fold significant increase in bacterial count across all biofilm types (Figure 3) (*p* < 0.05). Using T as a reference, the HF group showed similar bacterial counts in Pg (48 and 72 h) and Aa (72 h) (Figure 3) (*p* > 0.05), while other biofilms exhibited significantly higher bacterial counts (Figure 3) (*p* < 0.05). Using the T group as a baseline, the HN group showed significantly fewer bacteria in Pg (48 and 72 h) (Figure 3) (*p* < 0.05) and comparable bacterial counts in Pi (48 h) (Figure 3) (*p* > 0.05).

## 4. Discussion

The present study investigated the influence of material types and surface treatments on biofilm formation and bacterial survival across titanium and three variations of zirconia discs. In contrast to the titanium reference group (T), the M group in zirconia displayed significantly higher bacterial counts in all biofilm categories (*p* < 0.05), effectively rejecting the first null hypothesis. The facts revealed through this study showed that the artificially formed surface of zirconia using two types of laser systems had an effect on the formation of the biofilm.

Prior research has largely focused on bacterial CFUs without much consideration for the type of material used in implants [31,32]. The present study complements these works by showing that material type plays a critical role in bacterial proliferation. Earlier studies have often limited their investigation to single bacterial strains, whereas a multi-strain approach provides a more comprehensive view. Previous research has also reported on the efficacy of femtosecond lasers in biomaterial surface treatments [33], but the present study extends this by showing reduced bacterial adhesion in the laser-treated group (HN group). When comparing 48 h and 72 h, a significant increase in the CFU value was found overall. However, in the T group, no difference was observed in the CFU of Pg over time, as well as in the HN group. Through this, it was found that most Pg and Pi were formed in the T group and HN group within the initial 48 h. In particular, the HN group showed reduced bacterial formation in Pg bacteria compared to SLA-treated titanium implants, showing that it is an excellent alternative in terms of reducing bacterial formation. According to the literature, the growth of Pg and Pi can be suppressed by lactoferrin [34]. However, the factors contributing to the decrease in adhesion of Pg bacteria have not been identified. This is a limitation of this study and should be confirmed through further research. When comparing the HF group with the HN group, the CFU values were significantly lower in the HN group. When a grid is formed with the nanosecond laser rather than grooves being formed by the femtosecond laser, the roughness value increases, but it becomes hydrophobic and the level of bacterial adhesion decreases. According to the literature, it may be seen that the formation of biofilm decreases when a hydrophobic surface is formed through a laser. This means that patterns formed through the nanosecond treatment method can lower the formation of biofilms more than the femtosecond treatment method and clinically reduce the possibility of complications such as inflammation around implants. A previous study evaluated the biofilm growth of Aa, Pg, and Pi and biofilm formation on titanium and zirconia implants and suggested the potential of zirconia implants to alleviate peri-implantitis [35]. Another study investigated how different surface treatment methods, including femtosecond and nanosecond laser processing, affect the surface morphology, roughness, and biofilm formation of dental titanium implant discs, and found that in the early stages of biofilm growth, Aa levels were significantly higher in the hydrophilic treatment group than in the hydrophobic treatment group. Although lower in the treated group, after 72 h, the biofilm growth was similar in both groups [28].

Surface treatment methods significantly affected bacterial growth, substantiating the rejection of the second null hypothesis. This corroborates findings by other researchers who reported that surface modifications could alter bacterial adherence and biofilm formation rates [36,37]. The present study noted that the efficacy of surface treatments varied between bacterial strains, emphasizing the importance of a multi-strain approach for a more nuanced understanding. The formation of biofilms is significantly influenced by surface modification as well as material properties, and this was analyzed by evaluating surface contact angle and surface roughness in the present study.

Surface wettability can be classified into hydrophobic (90° < θ < 150°), hydrophilic (10° < θ < 90°), superhydrophobic (θ > 150°), and superhydrophilic (θ < 10°) [38]. Wettability, measured by contact angles, varied notably between the HN and HF groups, confirming that increased wettability influences bacterial adhesion. This supports observations made in previous studies but provides new insights into how this might differ among various bacterial strains. According to previous studies, the more hydrophobic the surface contact angle, the more hindered the formation of biofilm, but the hydrophilic nature has the opposite characteristic. However, the results reported that the surface contact angle did not have a significant effect when the biofilm was grown for a long period of time. Therefore, additional research is needed to evaluate biofilm formation over 72 h, as suggested in the present study. Moreover, to calculate the surface free energy, there must be values for contact angle measurements with water and diiodomethane on the surface. However, in this study, there was only a value for water, so it was impossible to analyze surface free energy.

The present study, while contributing valuable insights to the field of dental biomaterials and surface treatments, comes with a set of limitations that need to be acknowledged. One of the most significant caveats is that the study could not fully emulate the complex environment of the oral cavity, where multiple strains of bacteria interact with saliva and food debris under varying physical and chemical conditions. Our experiments focused on only three bacterial strains, which barely scratches the surface of the microbial diversity found in the human mouth.

Future research would benefit from extending the scope to include a broader array of bacterial strains, especially those implicated in common oral diseases like caries and periodontitis. In addition, adopting in vivo models would offer a more comprehensive understanding and realistic simulation of oral conditions. Beyond bacterial diversity, the study could be extended to account for other important variables such as pH fluctuations, temperature changes, and mechanical forces like chewing and brushing. This multi-variable approach could more accurately reflect the in-situ conditions dental implants would be exposed to, thus making the research more generalizable.

The application of femtosecond laser technology has been a growing trend in recent years, especially in biomaterial surface treatments. The appeal largely lies in its precision and the minimal heat it generates, which is of crucial importance in preserving the integrity of delicate biomaterials. Our study corroborated this, indicating that surface roughness was generally higher in the laser-treated groups compared to the mechanically treated ones. Specifically, we observed that surfaces treated with nanosecond lasers showed a significantly greater level of roughness than those treated with femtosecond lasers. According to previous studies, the surface formed through nanosecond treatment has increased surface roughness, but the wettability measurement results showed hydrophobic characteristics [39]. According to another study, it was confirmed that when a grid pattern was formed in ceramics, hydrophobic properties were exhibited [40]. This could have implications for various applications where different levels of surface roughness are desired. For instance, a greater level of surface roughness might be beneficial in applications where increased cell adhesion is required but could be detrimental in cases where bacterial adhesion needs to be minimized.

Interestingly, despite the higher surface roughness in laser-treated samples, bacterial adhesion was notably lower when compared to mechanically treated samples. This reduced bacterial adhesion showed levels that were comparable to our control (T) group, which is particularly encouraging. This suggests that zirconia discs, when treated with either femtosecond or nanosecond lasers, could serve as viable alternatives to the current gold standards in dental implants or other biomaterial applications. The reduced bacterial adhesion in laser-treated zirconia discs may pave the way for improved implant designs that could minimize the risk of bacterial infections—a common problem in dental implant failures. Thus, laser-treated surfaces may not only offer improved mechanical properties but also better biological responses, thereby enhancing the longevity and success rate of dental implants. Our results indicate a somewhat counterintuitive relationship between surface roughness and bacterial adhesion. Typically, one might expect that greater surface roughness would provide more surface area for bacterial adhesion. However, our findings show that increased roughness in laser-treated groups did not correlate with increased bacterial adhesion, suggesting that other factors, such as surface chemistry or wettability, may be at play. Further studies focusing on these attributes could provide a more nuanced understanding of this relationship.

## 5. Conclusions

This research evaluated the impact of surface treatments on titanium discs and three types of zirconia discs (machined, hydrophilic, hydrophobic) concerning biofilm formation and surviving bacterial count.

This study indicates that disc surface treatment plays a crucial role in biofilm formation, and specifically, the hydrophobic surface created through the nanosecond laser is effective in inhibiting the growth of the Pg bacterium. However, this inhibitory effect may vary depending on the type of bacteria. The results of the present study can contribute to the development of surface treatment techniques for implant materials used in medical clinical fields and provide a foundation for researching effective bacterial inhibition methods. Nevertheless, additional studies are required to validate these findings in a clinical environment.

## Figures and Tables

**Table 1:** Comparison of the surface contact angles in the different implant surface treatments.

Surface Treatment Type	Mean	SD	95% Confidence Interval		*p* *	Comparison **
			Lower	Upper		
T group	67.528	3.453	65.058	69.998	<0.001	A
M group	80.176	2.981	78.043	82.309		B
HF group	64.652	2.867	62.601	66.703		A
HN group	124.977	3.882	122.200	127.754		C

* Significant difference in the roughness of the different surface treatments determined on different surface types using the Kruskal–Wallis H test, *p* < 0.05. ** Significant differences among the different implant surface treatments are indicated by different capital letters using the Bonferroni correction, *p* < 0.05. The same letter indicates significance between the two groups.

**Table 2:** Comparison of roughness (µm) on the different implant surfaces.

Roughness Type	Surface Treatment Type	Mean	SD	95% Confidence Interval		*p* *	Comparison **
				Lower	Upper		
Ra	T group	1.645	0.111	1.507	1.783	<0.001	AB
	M group	1.081	0.396	0.590	1.573		A
	HF group	2.322	0.456	1.755	2.888		B
	HN group	27.105	5.643	20.097	34.112		C
Rz	T group	10.452	0.430	9.918	10.986	0.001	AB
	M group	4.437	0.310	4.053	4.822		A
	HF group	11.721	1.458	9.911	13.532		B
	HN group	133.379	20.770	107.590	159.169		C

* Significant difference of the roughness of the different implant surfaces determined using the Kruskal–Wallis H test, *p* < 0.05. ** Significant difference among the different implant surfaces is indicated by different capital letters using Bonferroni correction, *p* < 0.05. The same letters indicate significance between the two groups.

**Table 3:** Comparison of the viable bacterial counts (CFU/mL) of biofilms formed over 48 h on the different implant surfaces.

Biofilm Type	Surface Treatment Type	Mean	SD	95% Confidence Interval		*p* *	Comparison **
				Lower	Upper		
Aa	T group	4770.0	228.0	4486.9	5053.1	<0.001	A
	M group	10,395.0	206.5	10,138.6	10,651.4		B
	HF group	5600.0	176.8	5380.5	5819.5		C
	HN group	5010.0	65.2	4929.1	5090.9		A
Pg	T group	3928.0	193.3	3688.0	4168.0	<0.001	A
	M group	9860.0	232.9	9570.8	10,149.2		B
	HF group	4330.0	75.8	4235.8	4424.2		C
	HN group	3010.0	82.2	2908.0	3112.0		D
Pi	T group	4500.0	45.3	4443.8	4556.2	<0.001	A
	M group	13,060.0	119.4	12,911.8	13,208.2		B
	HF group	5740.0	147.5	5556.9	5923.1		C
	HN group	5400.0	79.1	5301.8	5498.2		D

* Significant difference of the viable bacterial counts on the different implant surfaces determined using the Kruskal–Wallis H test, *p* < 0.05. ** Significant difference among the different implant surfaces is indicated by different capital letters using Bonferroni correction, *p* < 0.05.

**Table 4:** Comparison of the viable bacterial counts (CFU/mL) of biofilms formed over 72 h on the different implant surface.

Biofilm Type	Surface Treatment Type	Mean	SD	95% Confidence Interval		*p* *	Comparison **	Comparison with 48 h
				Lower	Upper			*p* ***
Aa	T group	5353.6	256.6	5034.9	5672.3	<0.001	A	0.005
	M group	11,380.0	336.5	10,962.1	11,797.9		B	0.001
	HF group	5900.0	50.0	5837.9	5962.1		C	0.017
	HN group	5470.0	115.1	5327.1	5612.9		A	<0.001
Pg	T group	4119.6	73.2	4028.7	4210.5	<0.001	A	0.092
	M group	10,720.0	168.1	10,511.3	10,928.7		B	<0.001
	HF group	4470.0	57.0	4399.2	4540.8		C	0.012
	HN group	3190.0	65.2	3109.1	3270.9		D	0.005
Pi	T group	5039.2	209.3	4779.3	5299.1	<0.001	A	0.004
	M group	13,700.0	434.5	13,160.6	14,239.4		B	0.028
	HF group	6100.0	176.8	5880.5	6319.5		C	0.009
	HN group	5200.0	259.8	4877.4	5522.6		A	0.164

* Significant difference of the viable bacterial counts on the different implant surfaces determined using the Kruskal–Wallis H test, *p* < 0.05. ** Significant difference among the different implant surfaces is indicated by different capital letters using Bonferroni correction, *p* < 0.05. *** Significant difference between 48 and 72 h using the Wilcoxon signed-rank test, *p* < 0.05.

## Data Availability

The datasets used and/or analyzed during the current study are available from the corresponding author upon reasonable request.
